# Ribociclib with letrozole vs letrozole alone in elderly patients with hormone receptor-positive, HER2-negative breast cancer in the randomized MONALEESA-2 trial

**DOI:** 10.1007/s10549-017-4523-y

**Published:** 2017-10-22

**Authors:** Gabe S. Sonke, Lowell L. Hart, Mario Campone, Frans Erdkamp, Wolfgang Janni, Sunil Verma, Cristian Villanueva, Erik Jakobsen, Emilio Alba, Erik Wist, Anne M. Favret, Thomas Bachelot, Roberto Hegg, Paul Wheatley-Price, Farida Souami, Santosh Sutradhar, Michelle Miller, Caroline Germa, Howard A. Burris

**Affiliations:** 1 Department of Medical Oncology, Netherlands Cancer Institute/BOOG Study Center, Plesmanlaan 121, 1066 CX Amsterdam, The Netherlands; 2 Florida Cancer Specialists, 8931 Colonial Center Dr Suite 300, Fort Myers, FL 33905 USA; 3 Sarah Cannon Research Institute, 250 25th Avenue North #100, Nashville, TN 37203 USA; 4 Institut de Cancérologie de l’Ouest – René Gauducheau Centre de Recherche en Cancérologie, Boulevard Jacques Monod, Nantes, 44805 Saint-Herblain France; 5 Zuyderland Medical Center, Sittard-Geleen/Heerlen, 6162 BG Geleen, The Netherlands; 6 Universitätsklinikum Ulm, Prittwitzstraße 43, 89075 Ulm, Germany; 7 Tom Baker Cancer Centre, 1331 29th Street NW, Calgary, AB T2N 4N2 Canada; 8 University Hospital of Besançon, Hospital Jean-Minjoz, 25000 Besançon, France; 9 Lillebælt Hospital, Kabbeltoft 25, 7100 Vejle, Denmark; 10 Hospital Universitario Virgen de la Victoria, IBIMA, 29010 Málaga, Spain; 11 Oslo University Hospital, Ullernchausseen 70 Radiumhospitalet, 0379 Oslo, Norway; 12 Virginia Cancer Specialists PC, US Oncology, 8503 Arlington Blvd #400, Fairfax, VA 22031 USA; 13 Centre Léon Bérard, 28 Prom. Léa et Napoléon Bullukian, 69008 Lyon, France; 14 Hospital Pérola Byington Centro de Referência da Saúde da Mulher, Av. Brigadeiro Luís Antônio, 683-Bela Vista, São Paulo, SP 01317-000 Brazil; 15 Ottawa Hospital Research Institute, University of Ottawa, 501, Smyth Road, Ottawa, ON K1H 8L6 Canada; 16 Novartis Pharma AG, CH-4002 Basel, Switzerland; 17 Novartis Pharmaceuticals Corporation, One Health Plaza, East Hanover, NJ 07936 USA

**Keywords:** Breast cancer, CDK inhibitor, Ribociclib, Endocrine therapy, Elderly, Hormone receptor-positive

## Abstract

**Purpose:**

Determine the efficacy and safety of first-line ribociclib plus letrozole in elderly patients with HR+, HER2− advanced breast cancer.

**Methods:**

668 postmenopausal women with HR+, HER2− advanced breast cancer and no prior systemic therapy for advanced disease were enrolled in the Phase III MONALEESA-2 trial (NCT01958021); 295 patients were aged ≥ 65 years. Patients were randomized to ribociclib (600 mg/day; 3-weeks-on/1-week-off) plus letrozole (2.5 mg/day) or placebo plus letrozole until disease progression, unacceptable toxicity, death, or treatment discontinuation. The primary endpoint was PFS, which was evaluated in elderly (≥ 65 years) and younger (< 65 years) patients. Secondary endpoints included response rates and safety.

**Results:**

Ribociclib plus letrozole significantly improved PFS vs placebo plus letrozole in elderly (hazard ratio: 0.608; 95% CI 0.394–0.937) and younger patients (hazard ratio: 0.523; 95% CI 0.378–0.723). Overall response rates were numerically higher in the ribociclib vs placebo arm, regardless of age. Ribociclib plus letrozole was well tolerated in elderly patients, with the safety profile similar to the overall study population. Nausea, vomiting, alopecia, and diarrhea were > 10% more frequent in the ribociclib plus letrozole vs placebo plus letrozole arm in both subgroups; most events were grade 1/2. In elderly patients, grade 1/2 anemia and fatigue were > 10% more frequent in the ribociclib plus letrozole vs placebo plus letrozole arm and discontinuation rates were similar in both arms.

**Conclusions:**

Addition of ribociclib to letrozole is a valid therapeutic option for elderly patients with HR+, HER2− advanced breast cancer in the first-line setting.

## Introduction

Over 40% of patients with breast cancer in the United States are aged ≥ 65 years at diagnosis, with the median age for diagnosis 62 years [1]. Elderly patients are more likely to have hormone receptor-positive (HR+), human epidermal growth factor receptor 2-negative (HER2−) disease compared with younger patients; in the 2010 Surveillance, Epidemiology, and End Results (SEER) registry database, 58% of patients diagnosed with HR+, HER2− breast cancer were younger than 50 years, 63% were aged 50–64 years, and 68% of patients were aged 65 years or older [2].

First-line treatment options for elderly patients with HR+ advanced breast cancer are similar for younger patients [3], where endocrine therapy is recommended for most cases in the absence of visceral crisis [4–7]. Use of other therapies, such as chemotherapy or some targeted agents, are often delayed in elderly patients owing to their challenging side-effect profiles. Comorbidities such as hypertension, diabetes, and coronary disease are common in elderly patients and can impact therapy choice [3, 8].

Although elderly patients with HR+ breast cancer derive benefits from treatment with endocrine monotherapies, the development of endocrine resistance remains a problem in this patient population [3]. New therapeutic approaches that delay development of endocrine therapy resistance and take into account comorbid illnesses, functional status, quality of life, and geriatric assessments are needed to improve the medical care and survival outcomes of older patients with HR+ advanced breast cancer [3, 9]. Combination regimens targeting multiple signaling pathways, such as everolimus plus exemestane, have shown efficacy in elderly patients with disease previously resistant to endocrine monotherapies [3, 10], suggesting that combined targeted therapies may represent a valid treatment option in elderly patients. However, elderly patients are generally under-represented in clinical trials, which may reflect physician concerns regarding the impact of comorbidities or the increased risk of drug-induced toxicities [3]. The presence of multiple comorbidities and concerns regarding polypharmacy in elderly patients may result in a poorer overall physiologic function, reduced compliance, and complications due to drug–drug interactions; therefore physicians may opt for alternative treatment options [11, 12].

The phase III MONALEESA-2 study (clinicaltrials.gov, NCT01958021) reported that addition of the cyclin-dependent kinase (CDK)4/6 inhibitor ribociclib to letrozole is well tolerated and significantly improves progression-free survival (PFS) compared with letrozole alone as a first-line therapy for HR+, HER2− advanced breast cancer [13]. Here, we determine the safety and efficacy of ribociclib plus letrozole in elderly patients (≥ 65 years of age) enrolled in the MONALEESA-2 study.

## Methods

### Study design and participants

MONALEESA-2 is a phase III, international, randomized, double-blind, placebo-controlled study conducted at 223 centers in 29 countries worldwide. Eligible patients were postmenopausal women with HR+, HER2−, recurrent or metastatic breast cancer. Postmenopausal status was defined by prior bilateral oophorectomy, age ≥ 60, or age < 60 with amenorrhea for ≥ 12 months, and follicle-stimulating hormone and estradiol levels considered to be postmenopausal as per the local normal range. Patients had measurable disease (per Response Evaluation Criteria In Solid Tumors [RECIST] v1.1 [14]) or at least one predominantly lytic bone lesion; an Eastern Cooperative Oncology Group (ECOG) performance status of ≤ 1 [15]; and adequate bone marrow and organ function.

Patients were excluded if they had previously received a CDK4/6 inhibitor, or any systemic chemotherapy or endocrine therapy for advanced disease. Prior (neo)adjuvant therapy with a non-steroidal aromatase inhibitor was permitted if the disease-free interval was > 12 months. Patients with inflammatory breast cancer, central nervous system metastases, a history of cardiac disease or dysfunction (including QTcF > 450 ms at screening), or impaired gastrointestinal function that altered study drug absorption were not permitted. Concomitant medications with a known risk of prolonging QT interval or inducing torsades de pointes were prohibited.

The study was conducted in accordance with the Declaration of Helsinki, Good Clinical Practice guidelines, and applicable local regulations. The institutional review board at each participating center reviewed the protocol and subsequent amendments. All patients provided written informed consent before enrollment.

### Randomization and masking

Patients were randomized in a 1:1 ratio to receive ribociclib plus letrozole or placebo plus letrozole. Randomization was stratified by the presence of liver and/or lung metastases. No treatment crossover was permitted.

### Procedures

Patients received oral ribociclib (600 mg/day; 3-weeks-on/1-week-off in 28-day cycles) plus letrozole (2.5 mg/day; continuous schedule) or placebo plus letrozole until disease progression, unacceptable toxicity, death, or discontinuation for any other reason. Dose reductions for ribociclib (from 600 to 400 to 200 mg/day) were permitted to manage adverse events; letrozole dose reductions were not permitted. Patients who discontinued ribociclib/placebo were permitted to continue letrozole therapy.

Tumor assessments (computed tomography or magnetic resonance imaging) were conducted at screening, every 8 weeks during the first 18 months, every 12 weeks thereafter until disease progression (including for patients who discontinued due to reasons other than progressive disease), and at end of treatment. Imaging data were prospectively reviewed by an independent review committee blinded to treatment allocation.

### Outcomes

The primary endpoint was PFS, assessed by local investigators as per RECIST v1.1. The key secondary endpoint was overall survival. Other secondary endpoints included overall response rate, clinical benefit rate, and safety.

Adverse events were characterized and graded throughout the study as per National Cancer Institute Common Terminology Criteria for Adverse Events version 4.03 [16]. Centralized biochemical and hematologic laboratory tests were performed at screening, Day 15 of Cycle 1, and Day 1 of subsequent cycles until the end of treatment. Electrocardiogram (ECG) assessments were conducted at screening, Day 15 of Cycle 1, and Day 1 of Cycles 2 and 3 in all patients; after a protocol amendment, additional ECG assessments were performed on Day 1 of Cycles 4–9 in all patients, and on Day 1 of subsequent cycles in patients with a mean QTcF ≥ 481 ms at any time prior to Cycle 10. ECGs were reviewed by an independent central panel blinded to treatment allocation.

### Population pharmacokinetic analyses

Population pharmacokinetic analyses were performed to characterize the profile of ribociclib and evaluate the influence of covariates on pharmacokinetic parameters. A population pharmacokinetic model was developed using pharmacokinetic data collected from 208 patients who received 50–1200 mg ribociclib (134 patients received a starting dose of 600 mg/day [3-weeks-on/1-week-off]) across three phase I trials (NCT01898845, NCT01237236, and NCT01872260). In total, 4854 data points were collected and utilized for model development; 98% were in the first 2 cycles (up to 8 weeks after the first ribociclib dose). The model was developed in a stepwise manner; a base structural model was developed then expanded to a full covariate model with inclusion of all predefined parameter–covariate relations, and subsequently condensed to a final model with retention of only important covariates. Models were evaluated based on parameter estimates, diagnostic plots, and visual predictive checks. A total of 177 steady-state pharmacokinetic data points (pre-dose and 2 h post-dose) from a subset of patients in MONALEESA-2 (*n* = 93) were used to validate the predictive capability of the final model.

### Statistical analysis

Efficacy analyses were based on data from the full analysis set: all randomized patients were analyzed on an intent-to-treat basis. For analysis of the primary endpoint, Kaplan–Meier estimates were used to assess PFS in elderly and younger patients; hazard ratios and 95% confidence intervals (CIs) were estimated using a Cox proportional hazards model, stratified according to the presence or absence of liver or lung metastases. Assessment of PFS in the elderly subset was pre-specified in the statistical analysis plan. The cut-off of 65 years was defined according to international standards defining old age [17]. Other pre-specified subanalyses were carried out according to ECOG performance status, baseline metastatic sites (liver, lung, bone, etc.), prior hormonal therapy status, prior (neo)adjuvant chemotherapy status, *de novo* disease status, race, estrogen/progesterone receptor status, and selected biomarkers. To determine the consistency of treatment benefit for PFS across both subgroups, the interaction *p* value was obtained from the stratified Cox proportional hazard model that included treatment, subgroup, and treatment by subgroup interaction terms. For the secondary endpoints of overall response and clinical benefit rates, 95% CIs were computed based on normal approximation to the binomial method. Safety analyses were performed in patients who received at least 1 dose of a study regimen and had at least 1 postbaseline safety assessment.

## Results

### Patient characteristics and disposition

**Fig. 1:**
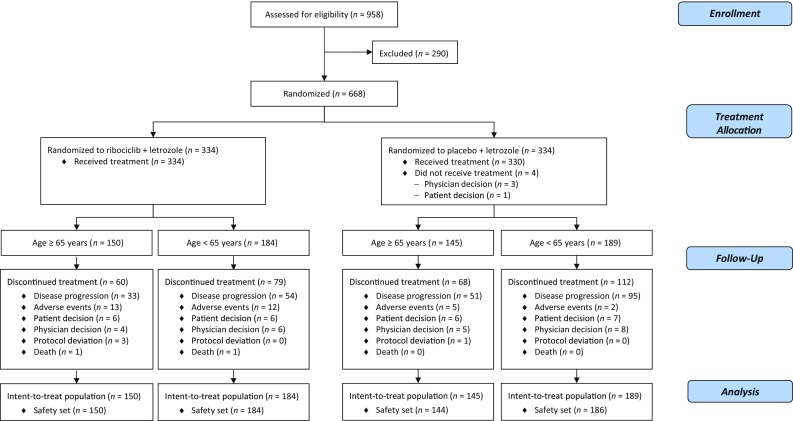
Trial profile (CONSORT diagram). *CONSORT* Consolidated Standards of Reporting Trials

In the MONALEESA-2 study, 668 patients were randomized to ribociclib plus letrozole (*n* = 334) and placebo plus letrozole (*n* = 334) between January 24, 2014 and March 24, 2015. Following international standards [17], the elderly population was defined as ≥ 65 years of age and included 295 patients. Distribution of patients ≥ 65 years and < 65 years was well balanced across the ribociclib plus letrozole vs placebo plus letrozole arms, with 150 (51%) vs 145 (49%), and 184 (49%) vs 189 (51%) patients, respectively (Table 1; Fig. 1). Patient characteristics, including site of metastases, were generally balanced across both treatment arms, and between patients aged ≥ 65 and < 65 years. Just over half (54%) of patients ≥ 65 years had an ECOG performance status of 0 compared with two-thirds (67%) of patients aged < 65 years (*p* = 0.001). A higher proportion of patients aged ≥ 65 years had an ECOG performance status of 1 in both treatment arms.

**Table 1:** Baseline characteristics according to patient age and treatment

Characteristic	Age ≥ 65 years (*n* = 295)		Age < 65 years (*n* = 373)	
	Ribociclib + letrozole (*n* = 150)	Placebo + letrozole (*n* = 145)	Ribociclib + letrozole (*n* = 184)	Placebo + letrozole (*n* = 189)
Median age, years (range)	70 (65–91)	71 (65–88)	55 (23–64)	56 (29–64)
ECOG performance status, *n* (%)				
0	80 (53)	79 (55)	125 (68)	123 (65)
1	70 (47)	66 (46)	59 (32)	66 (35)
Disease stage at initial diagnosis, *n* (%)				
I–II	70 (47)	66 (46)	83 (45)	89 (47)
III	22 (15)	24 (17)	36 (20)	38 (20)
IV	54 (36)	48 (33)	61 (33)	60 (32)
Disease stage at study entry, *n* (%)				
III	1 (1)	2 (1)	0	1 (1)
IV	149 (99)	143 (99)	184 (100)	188 (99)
Hormone receptor status, *n* (%)				
ER-positive	149 (99)	144 (99)	183 (99)	189 (100)
PgR-positive	121 (81)	122 (84)	150 (82)	156 (83)
Disease-free interval, *n* (%)				
De novo	54 (36)	52 (36)	60 (33)	61 (32)
Non-de novo (months)	96 (64)	93 (64)	124 (67)	128 (68)
≤ 12	1 (1)	4 (3)	3 (2)	6 (3)
> 12 to ≤ 24	6 (4)	4 (3)	8 (4)	11 (6)
> 24	89 (59)	84 (58)	113 (61)	111 (59)
Number of metastatic sites, *n* (%)				
0	1 (1)	0	1 (1)	1 (1)
1	43 (29)	51 (35)	57 (31)	66 (35)
2	54 (36)	40 (28)	64 (35)	63 (33)
≥ 3	52 (35)	54 (37)	62 (34)	59 (31)
Site of metastases, *n* (%)				
Breast	3 (2)	3 (2)	5 (3)	8 (4)
Bone	113 (75)	103 (71)	133 (72)	141 (75)
Bone only	35 (23)	33 (23)	34 (19)	45 (24)
Visceral^a^	91 (61)	85 (59)	106 (58)	111 (59)
Lymph nodes	57 (38)	59 (41)	76 (41)	64 (34)
Other^b^	19 (13)	11 (8)	16 (9)	11 (6)

*ECOG* Eastern Cooperative Oncology Group, *ER* estrogen receptor, *PgR* progesterone receptor

^a^Includes liver, lung, and other visceral sites

^b^Includes skin and bone marrow

At data cut-off (January 29, 2016), treatment was discontinued in 60 (40%) patients aged ≥ 65 years and 79 (43%) patients aged < 65 years receiving ribociclib plus letrozole, and in 68 (47%) and 112 (59%) patients aged ≥ 65 years and < 65 years receiving placebo plus letrozole, respectively (Fig. 1). The most common reason for treatment discontinuation was disease progression. Regardless of age, discontinuation due to disease progression occurred less frequently in the ribociclib plus letrozole arm vs the placebo plus letrozole arm. Additionally, a smaller proportion of elderly patients discontinued due to disease progression compared with younger patients (≥ 65 years vs < 65 years; ribociclib plus letrozole arm: 22% vs 29%; placebo plus letrozole arm: 35% vs 50%). The incidence of treatment discontinuation due to adverse events in the ribociclib plus letrozole vs placebo plus letrozole arms was similar in both subgroups (≥ 65 years: 9% vs 3%; < 65 years: 7% vs 1%).

### Efficacy

**Fig. 2:**
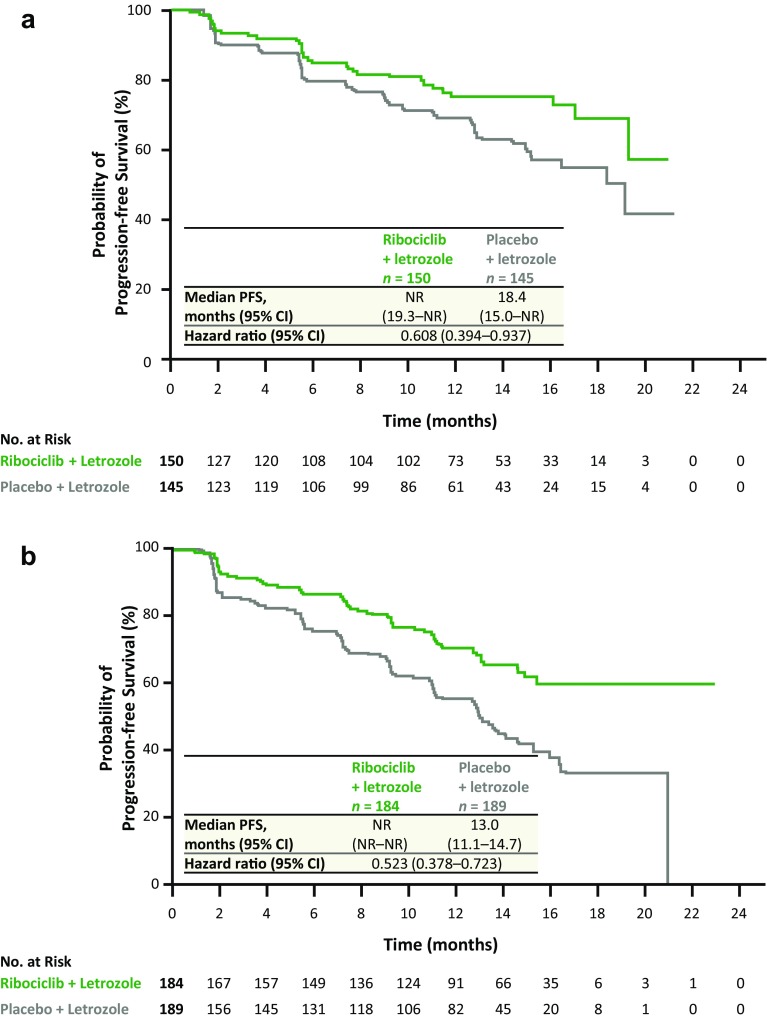
Kaplan–Meier analysis of locally assessed PFS with ribociclib plus letrozole vs placebo plus letrozole in patients aged ≥65 years (**a**) and <65 years (**b**). *CI* confidence interval, *HR* hazard ratio, *NR* not reached, *PFS* progression-free survival

Ribociclib plus letrozole significantly improved PFS compared with placebo plus letrozole, regardless of patient age. In patients ≥ 65 years, the risk reduction was 39% (hazard ratio: 0.608); in patients < 65 years old, the risk reduction was 48% (hazard ratio: 0.523; Fig. 2). Hazard ratios for both subgroups were in line with those seen in the full MONALEESA-2 patient population (hazard ratio: 0.56; 95% CI 0.43–0.72; *p* = 3.29 × 10^−6^ in all patients) [13]. There was no significant difference in ribociclib treatment effect between older and younger patients receiving ribociclib (interaction test *p* = 0.589). In both subgroups, median PFS was not reached in the ribociclib plus letrozole arm. In the placebo plus letrozole arm, median PFS was over 5 months longer in patients aged ≥ 65 years than in patients < 65 years (18.4 vs 13.0 months). Best overall responses are summarized in Table 2. In patients aged ≥ 65 years, overall response rates in the ribociclib plus letrozole arm vs placebo plus letrozole arm were 37% (95% CI 30–45) vs 31% (95% CI 24–39) and clinical benefit rates were 74% (95% CI 67–81) vs 75% (95% CI 67–82). Similarly, in patients aged < 65 years, the overall response rate and the clinical benefit rate were numerically higher in the ribociclib plus letrozole vs placebo plus letrozole arm (44% [95% CI 36–51] vs 25% [95% CI 19–31] and 84% [95% CI 79–90] vs 71% [95% CI 65–78], respectively). Overall survival data were immature at the time of analysis.

**Table 2:** Best overall response according to patient age and treatment

	Age ≥ 65 years (*n* = 295)		Age < 65 years (*n* = 373)	
	Ribociclib + letrozole (*n* = 150)	Placebo + letrozole (*n* = 145)	Ribociclib + letrozole (*n* = 184)	Placebo + letrozole (*n* = 189)
Confirmed BOR, *n* (%)				
CR	5 (3)	4 (3)	4 (2)	3 (2)
PR	51 (34)	41 (28)	76 (41)	44 (23)
SD	47 (31)	50 (35)	48 (26)	61 (32)
NCRNPD^a^	28 (19)	28 (19)	38 (21)	47 (25)
PD	7 (5)	14 (10)	12 (7)	26 (14)
Unknown	12 (8)	8 (6)	6 (3)	8 (4)
ORR^b^, *n* (%) [95% CI]	56 (37) [30–45]	45 (31) [24–39]	80 (44) [36–51]	47 (25) [19–31]
CBR^c^, *n* (%) [95% CI]	111 (74) [67–81]	108 (75) [67–82]	155 (84) [79–90]	135 (71) [65–78]

*BOR* best overall response, *CBR* clinical benefit rate, *CR* complete response, *NCRNPD* neither complete response nor progressive disease, *ORR* overall response rate, *PR* partial response, *RECIST* Response Evaluation Criteria In Solid Tumors, *SD*, stable disease

^a^NCRNPD was evaluated only among patients who had no measurable disease at baseline, according to RECIST v1.1

^b^ORR is defined as the proportion of patients with CR + PR as assessed by local investigators using RECIST v1.1

^c^CBR is defined as the proportion of patients with CR + PR + SD/NCRNPD (lasting ≥ 24 weeks) as assessed by local investigators using RECIST v1.1

### Safety and tolerability

The safety profile in the ribociclib plus letrozole arm was largely consistent across both age groups and was similar to that observed in the overall study population [13]. Median duration of exposure to ribociclib, placebo, or letrozole was comparable (12–13 months) across treatment arms and between age groups. The most common adverse events were hematologic and included neutropenia, leukopenia, and anemia (Table 3). Neutropenia was uncomplicated, with febrile neutropenia reported in 3 patients ≥ 65 years of age and in 2 patients aged < 65 years in the ribociclib plus letrozole arm. No patients in the placebo plus letrozole arm experienced febrile neutropenia.

**Table 3:** Adverse events (≥ 15% of patients in any arm) regardless of relationship to study drugs in patients aged ≥ 65 and < 65 years

Adverse event, *n* (%)	Age ≥ 65 years (*n* = 294)				Age < 65 years (*n* = 370)			
	Ribociclib + letrozole (*n* = 150)		Placebo + letrozole (*n* = 144^a^)		Ribociclib + letrozole (*n* = 184)		Placebo + letrozole (*n* = 186^a^)	
Grade	All-grade	Grade 3/4	All-grade	Grade 3/4	All-grade	Grade 3/4	All-grade	Grade 3/4
Total	148 (99)	130 (87)	139 (97)	56 (39)	181 (98)	141 (77)	181 (97)	52 (28)
Neutropenia^b^	111 (74)	90 (60)	7 (5)	0	137 (75)	108 (59)	10 (5)	3 (2)
Nausea	80 (53)	4 (3)	42 (29)	1 (1)	92 (50)	4 (2)	52 (28)	1 (1)
Diarrhea	61 (41)	3 (2)	37 (26)	1 (1)	56 (30)	1 (1)	36 (19)	2 (1)
Fatigue	55 (37)	3 (2)	35 (24)	2 (1)	67 (36)	5 (3)	64 (34)	1 (1)
Vomiting	53 (35)	6 (4)	27 (19)	1 (1)	45 (25)	6 (3)	24 (13)	2 (1)
Alopecia	49 (33)	0	25 (17)	0	62 (34)	0	26 (14)	0
Leukopenia^c^	46 (31)	31 (21)	5 (4)	1 (1)	64 (35)	39 (21)	8 (4)	1 (1)
Anemia^d^	39 (26)	2 (1)	9 (6)	2 (1)	24 (13)	2 (1)	6 (3)	2 (1)
Constipation	38 (25)	2 (1)	23 (16)	0	45 (25)	2 (1)	40 (22)	0
Arthralgia	37 (25)	1 (1)	40 (28)	2 (1)	54 (29)	2 (1)	55 (30)	1 (1)
Decreased appetite	34 (23)	4 (3)	25 (17)	0	28 (15)	1 (1)	25 (13)	1 (1)
Cough	29 (19)	0	28 (19)	0	36 (20)	0	31 (17)	0
Peripheral edema	29 (19)	0	17 (12)	0	22 (12)	0	17 (9)	0
Hypertension	28 (19)	23 (15)	28 (19)	25 (17)	20 (11)	10 (5)	21 (11)	11 (6)
Rash^e^	28 (19)	1 (1)	12 (8)	0	39 (21)	2 (1)	15 (8)	0
UTI^f^	28 (19)	2 (1)	15 (10)	0	21 (11)	0	26 (14)	0
Headache	27 (18)	1 (1)	21 (15)	0	47 (26)	0	42 (23)	1 (1)
Liver enzyme elevation^g^	26 (17)	14 (9)	9 (6)	3 (2)	34 (19)	18 (10)	9 (5)	5 (3)
ALT increased	24 (16)	14 (9)	6 (4)	0	28 (15)	17 (9)	7 (4)	4 (2)
AST increased	22 (15)	6 (4)	7 (5)	3 (2)	28 (15)	13 (7)	5 (3)	1 (1)
Asthenia	25 (17)	2 (1)	21 (15)	2 (1)	18 (10)	1 (1)	17 (9)	0
Back pain	23 (15)	2 (1)	30 (21)	1 (1)	43 (23)	5 (3)	28 (15)	0
Hot flush	22 (15)	1 (1)	27 (19)	0	48 (26)	0	51 (27)	0

*ALT* alanine aminotransferase, *AST* aspartate aminotransferase, *UTI* urinary tract infection

^a^Four patients in the placebo plus letrozole arm did not receive study treatment

^b^Neutropenia also includes ‘neutrophil count decreased’ and ‘granulocytopenia’

^c^Leukopenia also includes ‘white blood cell count decreased’

^d^Anemia also includes ‘anemia macrocytic’ and ‘hemoglobin decreased’

^e^Rash includes ‘maculopapular rash’

^f^UTI includes ‘cystitis’ and ‘escherichia UTI’

^g^Liver enzyme elevation includes increases in ALT, AST, and bilirubin levels

The most frequent non-hematologic adverse events included nausea, fatigue, alopecia, vomiting, and diarrhea; events were predominantly grade 1 or grade 2. In both age groups, nausea, alopecia, diarrhea, and vomiting were increased by 10% in the ribociclib plus letrozole over the placebo plus letrozole arm. A greater than 10% increase in the incidence of fatigue in the ribociclib plus letrozole over the placebo plus letrozole arm was observed in elderly patients. Incidence rates of anemia, hypertension, and asthenia were higher in elderly patients, irrespective of treatment arm. In the ribociclib plus letrozole arm, 1 patient aged ≥ 65 years with cardiac abnormalities, a cardiac assistance device, and a QTcF > 450 ms at baseline experienced a QTcF prolongation of > 500 ms, which resolved without the need for dose modification.

Adverse events were managed effectively by dose interruptions or reductions. In patients aged ≥ 65 years, 106 (71%) and 79 (53%) patients experienced ribociclib dose interruptions and reductions due to adverse events, respectively. Similarly, in patients aged < 65 years, 121 (66%) and 90 (49%) experienced ribociclib dose interruptions and reductions due to adverse events, respectively. Neutropenia was the most common adverse event leading to dose interruptions and reductions in both age groups. Despite dose modifications, the dose intensity of ribociclib was maintained at 86 and 90% in patients aged ≥ 65 and < 65 years, respectively.

### Pharmacokinetics

The median age of patients included in the population for pharmacokinetic model development was 60 years (range 23–82). The distribution of age was adequate to evaluate the effect of age on the pharmacokinetic profile of ribociclib; age distribution was as follows: < 40 years (*n* = 10), ≥ 40 to < 50 years (*n* = 29), ≥ 50 to < 60 years (*n* = 61), ≥ 60 to < 70 years (*n* = 71), ≥ 70 to < 80 years (*n* = 36), and ≥ 80 years (*n* = 1). The covariate effect of age on ribociclib clearance was estimated to be 1.018 (95% CI 0.875–1.324), indicating statistical insignificance and limited clinical importance.

## Discussion

Novel treatments that enhance the effectiveness of endocrine therapy and delay development of resistance are urgently needed in HR+ advanced breast cancer. Over two-thirds of patients diagnosed with HR+, HER2− breast cancer are aged 65 years or older. The need for novel treatment options is particularly relevant for elderly patients given the high incidence of pre-existing comorbidities and the perceived risk of more aggressive treatment options, such as certain chemotherapy regimens [11]. Dysregulation of the cyclin D–CDK4/6 inhibitor of CDK4 (INK4)–retinoblastoma (Rb) pathway in breast cancer cells has been associated with endocrine therapy resistance [18], and preclinical studies in HR+ breast cancer models have demonstrated improved efficacy when CDK4/6 inhibitors are combined with endocrine therapy [19–22]. Results from the MONALEESA-2 study demonstrated that dual blockade of the CDK4/6 and estrogen receptor pathways improves clinical outcomes in patients with HR+, HER2− advanced breast cancer; first-line ribociclib plus letrozole significantly prolonged PFS compared with letrozole alone (hazard ratio: 0.556; 95% CI 0.429–0.720; *p* = 3.29 × 10^−6^) [13]. Hematologic adverse events are commonly observed with CDK4/6 inhibitor therapy, including ribociclib [13, 23–25]. Given the reduced hematopoietic reserves in elderly patients, evaluation of ribociclib therapy in this patient population is particularly pertinent [26].

In the current pre-specified analysis of the MONALEESA-2 trial, ribociclib plus letrozole demonstrated clinical efficacy and manageable tolerability in elderly patients with HR+, HER2− advanced breast cancer. Ribociclib PFS benefit was maintained both in elderly and younger patients, with no significant difference observed in ribociclib treatment benefit between the two subgroups, as demonstrated by an interaction test (*p* = 0.589). In both age groups, patients derived early clinical benefit from ribociclib plus letrozole, with separation of the PFS curves occurring from 8 weeks onwards. Overall response rates were numerically higher with ribociclib plus letrozole compared with placebo plus letrozole, regardless of patient age (37% vs 31% for patients aged ≥ 65 years and 44% vs 25% in patients aged < 65 years). Other CDK4/6 inhibitor-based regimens have also demonstrated efficacy in elderly patients [23, 27], further supporting CDK4/6 inhibitors as a valuable treatment option in elderly patients with HR+ advanced breast cancer.

The observation that median PFS with placebo plus letrozole was over 5 months longer in patients aged ≥ 65 years compared with patients aged < 65 years, despite a similar distribution of metastatic site involvement between the age groups, supports previous suggestions that elderly patients may have a more indolent course of disease [11, 28]. Several reasons for increased incidence of indolent tumors in elderly patients have been proposed, including presentation of more aggressive disease in the younger patient population and/or poor circulation resulting in reduced oxygenation and limited cell proliferation in elderly patients [11]. However, in the MONALEESA-2 trial baseline Ki67 levels were similar in both age groups and across treatment arms (data not shown). Additionally, in elderly patients, there is a higher prevalence of the more indolent luminal A subtype compared with aggressive luminal B tumors [29, 30]. Despite the improvement in PFS observed for both age subgroups receiving ribociclib, the reason for there being less improvement in the overall response rate and clinical benefit rate in elderly patients vs younger patients receiving ribociclib plus letrozole may be that elderly patients have a more indolent disease vs younger patients. The numerically longer PFS observed in elderly vs younger patients receiving placebo plus letrozole highlights the importance of identifying biomarkers that may predict which patients are more likely to derive benefit from CDK4/6 inhibitor-based regimens compared with single-agent endocrine therapies. To date, exploratory subgroup analyses of CDK4/6 pathway-related biomarkers, such as Rb protein, cyclin D1, and p16, have demonstrated that the combination of CDK4/6 inhibitors plus letrozole consistently improves PFS, irrespective of biomarker status [31, 32]. Additional biomarker analyses for ribociclib are ongoing.

Ribociclib plus letrozole was well tolerated in elderly patients, with no new safety concerns raised and a safety profile comparable to that observed in the overall MONALEESA-2 patient population [13]. The safety profile in elderly patients was similar to that observed in younger patients, despite an increased proportion of elderly patients in the ribociclib plus letrozole arm presenting with an ECOG performance status of 1. As expected, hematologic adverse events were more common with ribociclib plus letrozole than placebo plus letrozole across both age groups. Interestingly, despite age being a known risk factor for neutropenia [33, 34], the incidence of neutropenia was comparable in patients aged ≥ 65 years and patients aged < 65 years in contrast to chemotherapy regimens, which often increase the risk of neutropenia in elderly patients [11]. Anemia was reported more frequently in elderly patients than younger patients in both treatment arms, even though baseline hemoglobin values were similar in elderly and younger patients. This is perhaps unsurprising, given that the incidence of anemia typically increases with age [35, 36]. However, despite the increased incidence of anemia in elderly patients receiving ribociclib plus letrozole, the frequency of grade 3 or grade 4 anemia was low. The incidence of fatigue in elderly patients was increased by more than 10% in the ribociclib plus letrozole arm vs the placebo plus letrozole arm, although these were predominantly grade 1 or grade 2 events. The incidence of both liver enzyme elevations and QT prolongation was similar across subgroups; both events were reversible and managed by dose interruptions and reductions, and there were no clinical consequences of these events in elderly patients. The overall incidence rates of dose interruptions and reductions were comparable in both age groups. The similar incidence of neutropenia and QT prolongation across age groups is consistent with the lack of an age effect on ribociclib exposure, as shown by the population pharmacokinetic analysis.

In conclusion, data from the phase III, randomized MONALEESA-2 study indicate that first-line ribociclib plus letrozole is effective in elderly patients with HR+ advanced breast cancer. Addition of ribociclib to letrozole is associated with a manageable tolerability profile in elderly patients, further supporting combined targeted approaches as a valid therapeutic option in this patient population.

## References

[1] Howlader N, Noone A, Krapcho M et al (2016) SEER cancer statistics review, 1975–2013. National Cancer Institute. http://seer.cancer.gov/csr/1975_2013/. Accessed 24 Aug 2017

[2] Howlader N, Altekruse S, Li C, Chen V, Clarke C, Ries L (2014). US incidence of breast cancer subtypes defined by joint hormone receptor and HER2 status. J Natl Cancer Inst.

[3] Riseberg D (2015). Treating elderly patients with hormone receptor-positive advanced breast cancer. Clin Med Insights Oncol.

[4] Rugo H, Rumble R, Macrae E (2016). Endocrine therapy for hormone receptor-positive metastatic breast cancer: American Society of Clinical Oncology guideline. J Clin Oncol.

[5] Cardoso F, Costa A, Senkus E (2016). 3rd ESO-ESMO international consensus guidelines for advanced breast cancer (ABC 3). Ann Oncol.

[6] Cardoso F, Costa A, Senkus E (2017). 3rd ESO-ESMO international consensus guidelines for advanced breast cancer (ABC 3). Breast.

[7] National Comprehensive Cancer Network. NCCN Clinical Practice Guidelines in Oncology. Breast Cancer, version 2.2017. http://www.nccn.org. Accessed 24 Aug 2017

[8] Spano J, Falandry C, Chaibi P, Freyer G (2011). Current targeted therapies in breast cancer: clinical applications in the elderly woman. Oncologist.

[9] Owusu C, Berger N (2014). Comprehensive geriatric assessment in the older cancer patient: coming of age in clinical cancer care. Clin Pract (Lond).

[10] Pritchard K, Burris HA, Ito Y (2013). Safety and efficacy of everolimus with exemestane vs. exemestane alone in elderly patients with HER2-negative, hormone receptor-positive breast cancer in BOLERO-2. Clin Breast Cancer.

[11] Tesarova P (2013). Breast cancer in the elderly-should it be treated differently?. Rep Pract Oncol Radiother.

[12] Balducci L, Goetz-Parten D, Steinman M (2013). Polypharmacy and the management of the older cancer patient. Ann Oncol.

[13] Hortobagyi G, Stemmer S, Burris H (2016). Ribociclib as first-line therapy for HR-positive, advanced breast cancer. N Engl J Med.

[14] Eisenhauer EA, Therasse P, Bogaerts J (2009). New response evaluation criteria in solid tumours: revised RECIST guideline (version 1.1). Eur J Cancer.

[15] Oken MM, Creech RH, Tormey DC (1982). Toxicity and response criteria of the Eastern Cooperative Oncology Group. Am J Clin Oncol.

[16] National Cancer Institute. Cancer Therapy Evaluation Program. Common Terminology Criteria for Adverse Events (CTCAE) v4.0. http://ctep.cancer.gov/protocolDevelopment/electronic_applications/ctc.htm. Accessed 1 June 2017

[17] World Health Organization. Health statistics and information systems. Proposed working definition of an older person in Africa for the MDS Project. http://www.who.int/healthinfo/survey/ageingdefnolder/en/. Accessed 10 Aug 2017

[18] Thangavel C, Dean JL, Ertel A (2011). Therapeutically activating RB: reestablishing cell cycle control in endocrine therapy-resistant breast cancer. Endocr Relat Cancer.

[19] O’Brien N, Di Tomaso E, Ayala R et al (2014) In vivo efficacy of combined targeting of CDK4/6, ER and PI3K signaling in ER+ breast cancer. In: Proceedings of the 105th Annual Meeting of the American Association for Cancer Research: Abstract 4756

[20] Parasuraman S, Caponigro G, Loo A (2014). LEE011, a potent and selective CDK4/6 inhibitor, under preclinical and clinical investigation. Targ Anticancer Ther Congress: Oral Present.

[21] Finn RS, Dering J, Conklin D (2009). PD 0332991, a selective cyclin D kinase 4/6 inhibitor, preferentially inhibits proliferation of luminal estrogen receptor-positive human breast cancer cell lines in vitro. Breast Cancer Res.

[22] Rugo HS, Vidula N, Ma C (2016). Improving response to hormone therapy in breast cancer: new targets, new therapeutic options. Am Soc Clin Oncol Educ Book.

[23] Finn RS, Martin M, Rugo HS (2016). Palbociclib and letrozole in advanced breast cancer. N Engl J Med.

[24] Sherr CJ, Beach D, Shapiro GI (2015). Targeting CDK4 and CDK6: from discovery to therapy. Cancer Discov.

[25] Asghar U, Witkiewicz AK, Turner NC, Knudsen ES (2015). The history and future of targeting cyclin-dependent kinases in cancer therapy. Nat Rev Drug Discov.

[26] Balducci L, Hardy C, Lyman C (2000). Hemopoietic reserve in the older cancer patient: clinical and economical considerations. Cancer Control.

[27] Cristofanilli M, Turner NC, Bondarenko I (2016). Fulvestrant plus palbociclib versus fulvestrant plus placebo for treatment of hormone-receptor-positive, HER2-negative metastatic breast cancer that progressed on previous endocrine therapy (PALOMA-3): final analysis of the multicentre, double-blind, phase 2 randomised controlled trial. Lancet Oncol.

[28] Yancik R, Ries L, Yates J (1989). Breast cancer in aging women. A population-based study of contrasts in stage, surgery, and survival. Cancer.

[29] McGuire A, Brown J, Malone C (2015). Effects of age on the detection and management of breast cancer. Cancers (Basel).

[30] Tran B, Bedard P (2011). Luminal-B breast cancer and novel therapeutic targets. Breast Cancer Res.

[31] Andre F, Stemmer S, Campone M et al (2017) Ribociclib + letrozole for first-line treatment of HR+, HER2− advanced breast cancer: efficacy by baseline tumor markers. In: AACR-NCI-EORTC International Conference on Molecular Targets and Cancer Therapeutics: Abstract CT045

[32] Finn R, Jiang Y, Rugo H (2016). Biomarker analyses from the phase 3 PALOMA-2 trial of palbociclib (P) with letrozole (L) compared with placebo (PLB) plus L in postmenopausal women with ER+/HER2− advanced breast cancer (ABC). Ann Oncol.

[33] López-Pousa A, Rifà J, Casas de Tejerina A (2010). Risk assessment model for first-cycle chemotherapy-induced neutropenia in patients with solid tumors. Eur J Cancer Care.

[34] Lustberg M (2014). Management of neutropenia in cancer patients. Clin Adv Hematol Oncol.

[35] Rao A, Cohen H (2004). Symptom management in the elderly cancer patient: fatigue, pain, and depression. J Natl Cancer Inst Monogr.

[36] Patel K (2008). Epidemiology of anemia in older adults. Semin Hematol.

